# Low-Density and High-Performance Fiber-Reinforced PP/POE Composite Foam via Irradiation Crosslinking

**DOI:** 10.3390/polym16060745

**Published:** 2024-03-08

**Authors:** Hongfu Li, Tianyu Wang, Changwei Cui, Yuxi Mu, Kangmin Niu

**Affiliations:** School of Materials Science and Engineering, University of Science and Technology Beijing, Beijing 100083, China

**Keywords:** polypropylene, melt strength, thermoplastic foam, irradiation crosslinking, fiber composite, mechanical property

## Abstract

This study addresses the challenge of achieving foam with a high expansion ratio and poor mechanical properties, caused by the low melt viscosity of semi-crystalline polypropylene (PP). We systematically employ a modification approach involving blending PP with polyolefin elastomers (POE), irradiation crosslinking, and fiber reinforcement to prepare fiber-reinforced crosslinked PP/POE composite foam. Through optimization and characterization of material composition and processing conditions, the obtained fiber-reinforced crosslinked PP/POE composite foam exhibits both low density and high performance. Specifically, at a crosslinking degree of 12%, the expansion ratio reaches 16 times its original value, and a foam density of 0.057 g/cm^3^ is reduced by 36% compared to the non-crosslinked PP/POE system with a density of 0.089 g/cm^3^. The density of the short-carbon-fiber-reinforced crosslinked sCF/PP/POE composite foam is comparable to that of the crosslinked PP/POE system, but the tensile strength reaches 0.69 MPa, representing a 200% increase over the crosslinked PP/POE system and a 41% increase over the non-crosslinked PP/POE system. Simultaneously, it exhibits excellent impact strength, tear resistance, and low heat shrinkage. Irradiation crosslinking is beneficial for enhancing the melt strength and resistance to high temperature thermal shrinkage of PP/POE foam, while fiber reinforcement contributes significantly to improving mechanical properties. These achieve a good complementary effect in low-density and high-performance PP foam modification.

## 1. Introduction

Due to the ease of synthesis, excellent comprehensive performance, and low cost of polypropylene (PP), it has become one of the most widely used plastics globally, and is extensively applied in various aspects of our daily lives [1]. For instance, PP can achieve lightweight design and high specific strength structural application through foaming processes, while possessing characteristics such as thermal insulation. Particularly driven by the increasing demand in recent years, there is a significant need for PP in emerging fields, like sandwich structures of automotive structures, interiors, food packaging, and other fields [1,2,3]. Enhancing the foaming expansion ratio and mechanical properties of PP foam to reduce the usage of chemically synthesized materials represents a crucial direction in the development of polymer foam applications under the impetus of a low carbon footprint.

However, due to the uniform chain structure and high crystallinity of PP, its low melt strength near foaming temperature makes it challenging to support the stable growth of high expansion ratio bubbles, leading to the occurrence of bubble rupture. For instance, Lee [4] investigated the relationship between the extrusion foaming ratio and processing temperature of PP. It was found that, during the foaming process, if the temperature is too high, the melt strength of PP significantly decreases, resulting in severe gas escape and potential damage to the bubble structure. On the other hand, if the temperature is too low, the melt strength becomes excessively high, hindering the growth of bubbles. The optimal foaming temperature for the system was identified as 190 °C, and it was emphasized that the foaming temperature needs to consider the impact of the system and foaming agent type. Burt [5] evaluated the foaming temperature window for PP and found a narrow temperature range of only 4 °C suitable for PP foaming. Therefore, to achieve PP foaming materials with a high expansion ratio, enhancing the melt strength of PP during the foaming process and expanding the operational temperature window are crucial technical challenges.

To enhance the melt strength of PP, various approaches such as blending with thermoplastic elastomers [6,7,8], introducing side-chain branching to PP [9,10], and crosslinking [11,12,13,14] can be employed. For instance, Wang and Gong [8,15] investigated blending modifications of PP and polyolefin elastomers (POE), revealing that POE exhibits higher melt strength, good flowability, lower melting temperature, compatibility with PP, and ease of processing. Additionally, incorporating POE can reduce the crystallinity of PP, which is advantageous for minimizing the sensitivity of melt strength to temperature changes, and enhancing the impact resistance of PP. Han [16] utilized a two-step method involving a foaming agent, crosslinking agent blending modification, and irradiation crosslinking to improve the melt strength of PP. Subsequently, foaming of the crosslinked PP was achieved using compression molding, resulting in PP foam with uniform pore distribution, small pore size, and excellent mechanical properties. Notably, physical crosslinking methods such as irradiation [17,18,19] are more environmentally friendly compared to chemical crosslinking methods. They effectively avoid the use of volatile peroxide initiators and chemical crosslinking agents like benzoyl peroxide (BPO), dicumyl peroxide (DCP), and triallyl isocyanurate (TAIC), aligning with development needs for a low carbon footprint and low VOC emissions [13].

However, the high foaming ratio results in a significant reduction in material density, and it is challenging to simultaneously maintain excellent mechanical properties. Well-dispersed particles like clay [20,21,22], talc [23,24], nano-CaCO_3_ [25], hollow molecular-sieve particles [26], CNT [27], and graphene [28] are generally used to enhance PP cell structures and mechanical properties. However, the reinforcing effect of particles is often limited. Fiber reinforcement has proven to be an effective method for enhancing the performance of polymer materials [29,30] and concrete [31,32]. In recent years, the concept of fiber reinforced polymer composites has gradually been applied to the research on the reinforcement and modification of high-performance polymer foams. Relevant results indicate that fiber parameters such as strength, length, and content can influence the thermal and mechanical properties of polymers, as well as their foaming behavior [33,34,35,36]. Sebaey [37,38] investigated the energy absorption behavior of polyurethane (PU) foam-filled carbon-fiber-reinforced polymer composite tubes under impact. Chuang [39] developed carbon-fiber- and glass-fiber-reinforced foamed PU composite multifunctional protective boards suitable for diversified environments. Kumar [40] emphasized the reinforcement of rigid polyurethane foam (RPUF) via the addition of glass fibers for diverse engineering applications. Mechanical properties were found to be improved with addition of GF content due to the increased foam density and decreased cell size. Kuranchie [41] reviewed the effect of natural fiber reinforcement on PU composite foams. Shen [42] carried out foaming analysis of polystyrene-carbon nanofiber nanocomposite. However, for PP polymer, research is primarily concentrated on fiber-reinforced non-foamed PP composites [43,44,45], with limited reports on attempts to obtain fiber-reinforced PP composite foam. Wang [46] attempted to enhance PP fine-celled foaming with the incorporation of short carbon fibers via melt compounding in a twin-screw extruder, followed by injection molding and foaming with supercritical CO_2_. The most uniform foam size distribution and cell morphology were achieved with 25 wt.% carbon fibers. Nonetheless, a large number of unfoamed regions could still be observed. Furthermore, a low foaming rate, indicated by a high foam density of 0.58 g/cm^3^, was achieved due to the increased melt viscoelasticity at high fiber content, which inhibited the foaming process. Cai [35] predicted the elastic moduli of the foamed glass-fiber-reinforced PP composite foam through experiments and by constructing a multilayer RVE model. Rachtanapun [47] investigated wood fiber reinforced PP/HDPE blend composites to determine the effects of processing condition, blend composition, and wood fiber content on the void fraction and cell morphology of the materials without involving the study of mechanical properties. Therefore, it is evident that there are still challenges in achieving a high foaming expansion ratio while maintaining high strength for fiber-reinforced PP.

Based on this, we simultaneously employed a blend modification approach using POE thermoplastic elastomer and physical irradiation crosslinking to enhance the melt strength of PP, aiming to produce a higher expansion ratio and low-density PP foam. Simultaneously, we utilized short glass fibers (sGF) and short carbon fibers (sCF) to enhance the mechanical properties of the PP foam. Through optimization of material composition, process conditions, and characterization assessments, we systematically investigated the influence of thermoplastic elastomers, crosslinked network structures, fiber types, and content on the foaming, thermal stability, and mechanical performance of PP, ultimately achieving the fabrication of low-density and high-performance fiber-reinforced PP/POE composite foam.

## 2. Materials and Methods

### 2.1. Materials

Isotactic polypropylene (PP), a brand of S131, with a molecular weight (M_w_) of 420,804 and a melt flow index of 1.3 g/10 min was supplied by Sumitomo Chemical Co., Ltd., Tokyo, Japan. Polyolefin elastomer (POE), a brand of 6202, exhibiting a Shore hardness of 64 A and a melt flow index of 9.1 g/10 min was obtained from ExxonMobil Corporation, Spring, TX, USA. The foaming agent ADC 271, primarily composed of azodicarbonamide, was sourced from Baerlocher GmbH, Unterschleißheim, Germany. Stearic acid was utilized as a lubricant during processing. The antioxidant 1076G was obtained from BASF. The nucleating agent TMA-3, identified as an α-type nucleating agent for improving the cell distribution of foaming materials, was procured from Anhui Xiangyun Rubber Plastic Co., Ltd., Hefei, China. Triallyl isocyanurate (TMPTMA), a brand of TMPTMA-P, was used as a crosslinking agent and was provided by Anhui Xiangyun Chemical Co., Ltd., Hefei, China. Short carbon fibers (sCF), specifically T300 with a length of 2 mm, were procured from Toray. Short glass fibers (sGF) with a length of 3 mm were supplied by Shanghai Lishuo Composite Materials Co., Ltd., Shanghai, China.

### 2.2. Preparation of PP/POE Blend Sheets Ready to Foam

**Figure 1:**
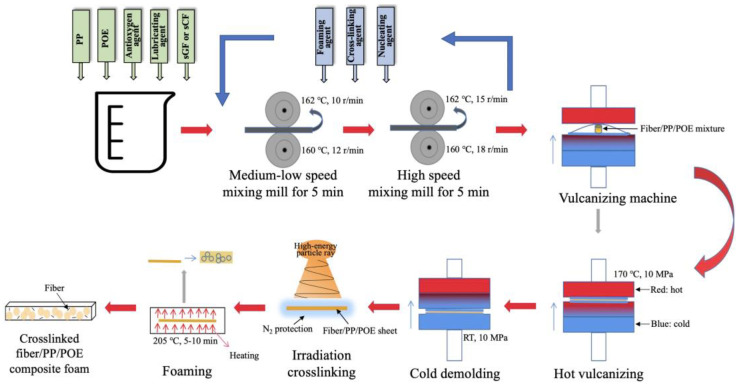
Diagram for the preparation of PP/POE and fiber-reinforced PP/POE blend sheet, irradiation crosslinking, and foaming processes.

In this study, a two-step compression molding foaming process was employed to prepare PP foam samples. This method requires low flowability of the polypropylene melt, making it particularly suitable for convenient operations when modifying the foaming system through blending, incorporating fibers, or introducing other inorganic fillers [48]. As shown in Figure 1, initially, according to the proportions listed in Table 1, a banbury mixer (XH-401CE-160, Guangdong Xihua Machinery Co., Ltd., Dongguan, China) was utilized to mix PP, POE, stearic acid, antioxidant, and either sCF or sGF at 160 °C for 5 min at medium speeds and high speeds, respectively, resulting in the preparation of a fiber-reinforced PP/POE premix. Subsequently, the foaming agent, nucleating agent, and crosslinking agent were added, followed by low-speed mixing for 5 min and high-speed mixing for an additional 5 min. The mixed material was then placed on a flat vulcanization bed, degassed at 170 °C for 1 min, and held under high pressure for 90 s to mold the pre-mixture into PP/POE sheets ready to foam.

### 2.3. Radiation Crosslinking and Foaming of PP/POE Blend Sheet

The premixed samples of PP/POE or fiber-reinforced PP/POE sheets were placed in plastic bags. After purging air and introducing nitrogen, the bags were sealed. An electron accelerator (GJ-2, Sichuan Atomic Energy Research Institute) was used to induce active radicals and crosslinking in PP by adjusting the irradiation dose settings to 2/4/8/10/15/20 kGy. The irradiated sheets were then heat treated at 120 °C for 2 h to eliminate residual free radicals generated during the irradiation process. Subsequently, the heat treated premix sheets were placed in a coating machine for foaming, with a foaming temperature of 200–220 °C and a dwell time of 5–7 min, resulting in the final production of crosslinked PP/POE blended foamed sheets.

### 2.4. Characterizations

The density of PP premix sheets and foams was measured using a solid density analyzer (KW-300Y). The foaming expansion ratio *V_f_* was determined by Formula (1):

(1) $$V_{f\;}=\frac{\rho_{u}}{\rho_{f}}$$

where *V_f_* represents the foaming expansion ratio, *ρ_u_* is the density of the unfoamed sample in g/cm^3^, and *ρ_f_* is the density of the foamed sheet in g/cm^3^.

The degree of crosslinking was assessed through gel content in PP. Approximately 0.2 g of the crosslinked and unfoamed sample was weighed and recorded as *W*. After the sample was wrapped in a copper mesh with a 200 mesh and dried at 80 °C for 30 min, the dried mass was recorded as *W_1_*. Following this, the sample was placed in 140 °C xylene and stirred reflux for 6 h. Finally, the PP mass, after removing the copper mesh and drying at 80 °C for 1 h, was recorded as *W_2_*. The gel content *G* was calculated using Formula (2):

(2) $$G=\left(1-\frac{W_{1}-W_{2}}{W}\right)\times 100\%$$

high temperature thermal shrinkage performance involved placing 20 mm × 20 mm × 4 mm samples in a 160 °C oven for 10 min. After the samples cooled to room temperature and were allowed to stand for 2 h, the changes in thickness before (*H_u_*) and after (*H_ρ_*) thermal treatment were measured, and the thermal stability parameter α was calculated according to Formula (3):

(3) $$\alpha =\frac{H_{u}-H_{\rho }}{H_{\rho }}$$

the dynamic rheological properties were tested using a rotational rheometer (MCR302). A parallel plate mold with a diameter of 35 mm and a 1 mm sample gap was used. The frequency was set from 0.1 to 100 rad/s, and the linear viscoelastic range strain was 2%. The storage modulus, loss modulus, viscosity, and tan δ of the polymer matrix were tested at 200 °C in a N_2_ environment. 

Tensile strength of the samples followed ASTM D638 standard, with sample dimensions of 200 mm × 15 mm × 4 mm and a stretching rate of 10 mm/min. Impact strength was evaluated using a simple beam impact tester (XJ-300) for specimens without notches, measuring 75 mm × 15 mm × 4 mm, with an impact speed of 2.9 m/s. Tear strength testing followed ASTM D 3574 standard, with a sample size of 150 mm × 20 mm × 4 mm, containing a triangular notch in the middle. The crosshead speed of grip separation was 500 mm/min.

Microscopic foam pore morphology was observed using a scanning electron microscope (SEM). Image J software v1.54 was employed for pore size and distribution statistics.

## 3. Results and Discussion

### 3.1. Irradiation Crosslinking Process Optimization of PP/POE Foam

#### 3.1.1. Crosslinking Degree

**Figure 2:**
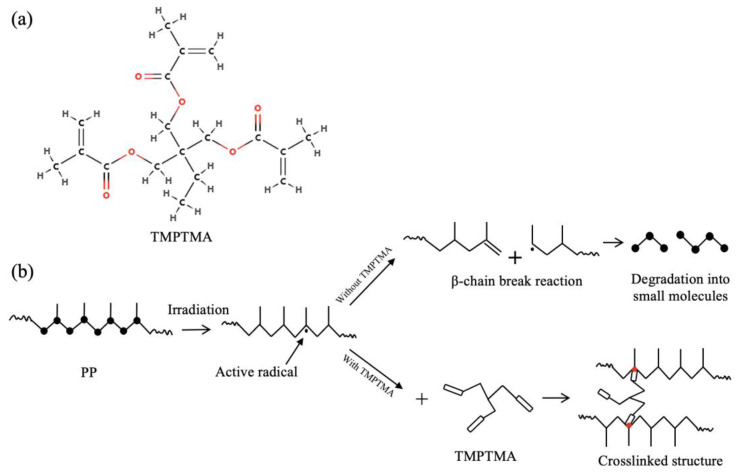
(**a**) Molecular structure of the crosslinking agent TMPTMA, (**b**) mechanism of PP chain scission and crosslinking induced by irradiation.

The mechanism of chemical structure crosslinking induced by irradiation in PP is illustrated in Figure 2. Under the influence of irradiation, high-energy particles excite PP to generate tertiary carbon radicals. In the absence of a crosslinking agent in the system, the generated tertiary carbon radicals tend to undergo β-scission reactions [49], leading to the main chain breakage of PP. This results in a decrease in molecular weight, and further decomposition into small molecules. The melt strength of these small molecules is lower, making it less favorable for foaming. However, when a crosslinking agent with double bonds and multifunctional groups is present in the PP system, its carboxyl functional group can effectively inhibit β-scission reactions under irradiation. The carboxyl functional group binds with tertiary carbon radicals, forming more stable radicals. These stable radicals can undergo coupling termination reactions with the remaining tertiary carbon radicals, forming a crosslinked structure or network structure. The addition of a crosslinking agent and irradiation dosage are the two main influencing parameters affecting the degree of crosslinking in PP [50]. The degree of crosslinking is generally characterized by the gel content of the material, where a higher gel content corresponds to a higher degree of crosslinking. In this study, Trimethylolpropane trimethacrylate (TMPTMA) was chosen as the crosslinking agent, primarily controlling the crosslinking reaction through its double bond structure, as depicted in Figure 2.

**Figure 3:**
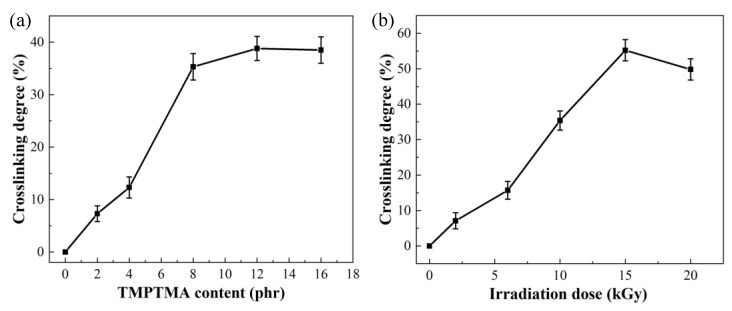
The change in crosslinking degree with variations in (**a**) crosslinking agent content and (**b**) irradiation dosage. (phr: an abbreviation for “Parts per Hundred”. In this study, it specifically means “with PP/POE as 100 parts, and the proportion of other materials relative to PP/POE in terms of parts”).

Figure 3a illustrates the relationship between the crosslinking degree and the amount of crosslinking agent at an irradiation dosage of 10 kGy. It is evident that, with a crosslinking agent content below 8 phr, the crosslinking degree increases with an increasing crosslinking agent. However, beyond 8 phr, the trend becomes more gradual. This is attributed to the excess tertiary carbon radicals generated in the system due to irradiation. As the content of the crosslinking agent increases, the crosslinking degree shows a positive correlation. However, the limited production of tertiary carbon radicals at 10 kGy can result in an insufficient supply of radicals to match the increased amount of crosslinking agent and facilitate additional crosslinking. At this point, the crosslinking degree reaches a peak, entering a plateau phase.

Figure 3b illustrates the relationship between crosslinking degree and irradiation dosage at a crosslinking agent content of 8 phr. It is evident that an initial increase in irradiation dosage significantly enhances the crosslinking degree. At an irradiation dosage of 15 kGy, the crosslinking degree reaches its maximum at 55%. Subsequently, with further increases in irradiation dosage, the crosslinking degree shows a declining trend. The observed decrease may be attributed to the accumulation of tertiary carbon radicals in the system with increasing irradiation dosage. However, since the crosslinking agent content in the system is fixed, the number of tertiary carbon radicals available for forming a crosslinked system with the crosslinking agent is limited. Excessive radicals can trigger the aforementioned β-scission reactions, causing PP to degrade into numerous small molecules and resulting in a decrease in gel content.

It can be concluded that the amount of the crosslinking agent and the irradiation dose jointly determine the PP/POE crosslinking degree. Achieving a high crosslinking degree requires a high irradiation dose and an adequate amount of crosslinking agent corresponding to the irradiation dose. Additionally, under the experimental conditions of this study, the crosslinking degree of the crosslinked PP/POE blend system can be regulated within a relatively broad range of 0–60%. Therefore, by adjusting the ratio of irradiation dose to crosslinking agent content, the crosslinking degree, melt strength, and flowability of the PP/POE blend can be controlled. This, in turn, allows for the control of the foaming behavior, mechanical properties, and thermal stability of PP foams.

#### 3.1.2. Foaming and Rheological Behaviors

**Figure 4:**
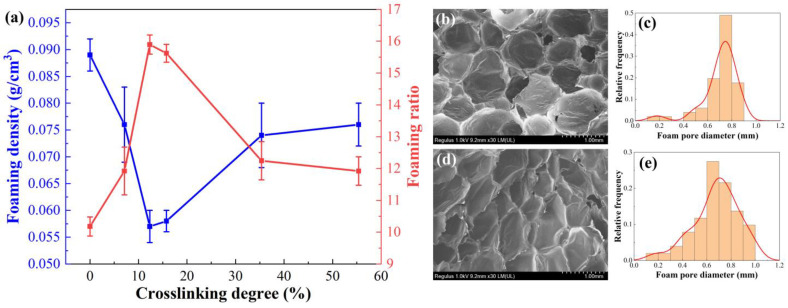
(**a**) The influence of crosslinking degree on foaming density and foaming expansion ratio, as well as the cellular morphology and pore size distribution at crosslinking degrees of (**b**,**c**) 12% and (**d**,**e**) 55%.

The crosslinking degree has a certain impact on the melt strength and flowability of the system during foaming, thereby influencing the foaming effect of the foam material. We employ different PP/POE premix sheets with varying crosslinking degrees and compare their lowest achieved foaming density to explore the influence of the crosslinking degree on the foaming effect. Figure 4 shows the relationship between the density and the crosslinking degree of PP/POE foam. It is evident that with an increase in the crosslinking degree, the density of the foamed material significantly decreases. Particularly, when the crosslinking degree reaches 12%, the density reaches its lowest point, with a foaming density of only 0.057 g/cm^3^ and a corresponding foaming ratio of 16. The density is much lower than that of non-crosslinked PP/POE blend and that reported in the literatures [13,25,46]. As the crosslinking degree continues to increase to 35%, the foaming density shows a noticeable increase and then maintains a constant plateau up to 55%, with a density of 0.076 g/cm^3^. However, the density is still lower than that of the non-crosslinked material, which has a density of 0.089 g/cm^3^. It is evident that the foaming density does not exhibit a purely positive correlation with the crosslinking degree. This is because, with an increase in the crosslinking degree within the system, the melt strength of the foaming system continues to improve. However, when the crosslinking degree is too high, such as 55%, the flowability of the melt is significantly restricted due to high viscosity, leading to insufficient expansion and growth of the bubbles, affecting both the size and quantity of the generated pores. Ultimately, this results in a decrease in the foaming ratio. Han reported that the melt viscosity drops remarkably above 2 kGy, decreasing to below the melt viscosity of the virgin PP [16]. Overall, a crosslinking degree of 12% demonstrates the best foaming effect for the system, achieving a foaming ratio of 16.

**Figure 5:**
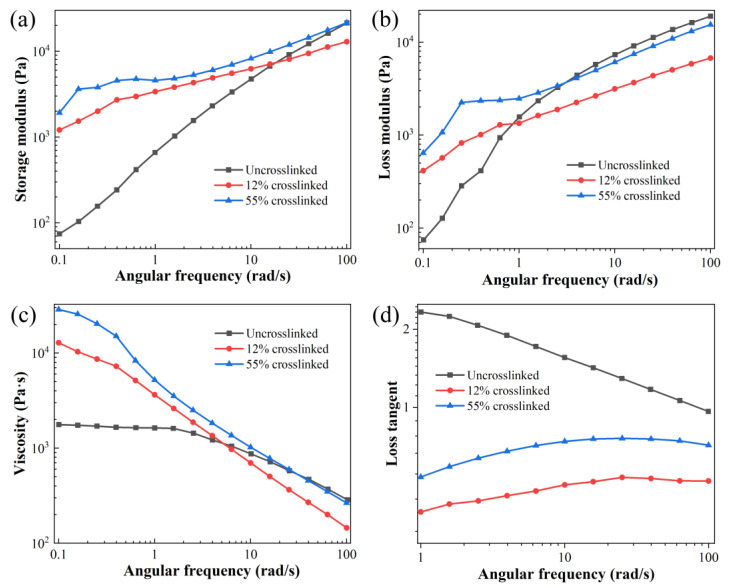
The impact of crosslinking degree on the melt properties of (**a**) storage modulus, (**b**) loss modulus, (**c**) viscosity, and (**d**) loss factor.

The impact of increasing crosslinking degree on the enhancement of melt strength in the PP foaming system and its influence on foam morphology can be elucidated through the rheological properties presented in Figure 5. Figure 5a illustrates the relationship between the storage modulus of different crosslinking degrees in the crosslinked PP/POE blend foaming matrix and shear rate. It can be observed that the storage modulus increases with the increment of shear rate, and the crosslinked PP/POE blended system with a higher crosslinking degree exhibits a larger storage modulus. Since the storage modulus at low shear viscosity reflects the elasticity of the melt, it is evident that the elasticity of the melt is significantly enhanced after the introduction of crosslinking modification, contributing to the growth of bubbles.

Figure 5b depicts the relationship between the loss modulus of the melt and shear rate. At low shear rates, the crosslinked PP/POE blended system has a higher loss modulus compared to the non-crosslinked blended system, which may be attributed to the formation of a crosslinked network in the crosslinked system, limiting the movement of the melt. Figure 5c shows that the viscosity of the PP/POE blended system exhibits a typical shear-thinning phenomenon due to the gradual orientation of the POE and PP polymer chains during oscillatory shear. At low shear rates, both the storage modulus and viscosity of the crosslinked system increase significantly when compared to the non-crosslinked system. As the shear rate increases, they begin to become indistinguishable. However, due to the constraints imposed by the crosslinked structure, the viscosity of the crosslinked PP/POE blended system is higher than that of the non-crosslinked system.

Furthermore, by examining the loss factor, the ratio of the loss modulus to the storage modulus, it is evident that the loss factor of the crosslinked system is significantly smaller than that of the non-crosslinked system (Figure 5d). Simultaneously, the loss factor of the crosslinked system is less than one, indicating that the elastic modulus in the crosslinked system is significantly higher than the loss modulus, and the melt exhibits better elasticity. This implies that the crosslinked PP/POE system can to some extent prevent the merging and rupture of bubbles. Additionally, it can be observed that when the crosslinking degree reaches 55%, the loss factor of the system slightly increases. This also explains the reduction in pore size and uneven distribution at a 55% crosslinking degree: the increased viscosity in the viscoelasticity leads to poor melt flowability, restricting the flow of the melt and causing insufficient growth dynamics of pore size. Therefore, from the perspective of rheological behavior, crosslinking modification shows a significant improvement in the foaming effect of the system. However, there is a peak value, and an excessively high crosslinking degree is unfavorable for the growth of bubbles.

#### 3.1.3. Thermal and Mechanical Properties

##### Thermal Shrinkage Performance

**Figure 6:**
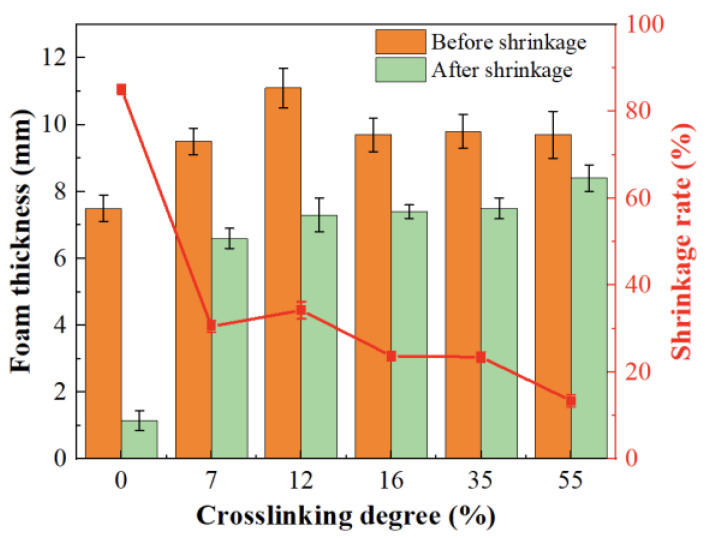
The thermal shrinkage rate of PP/POE foams as a function of crosslinking degree.

Figure 6 illustrates the dimensional changes of foam materials with different crosslinking degrees after high temperature treatment at 160 °C for 10 min. It is evident that with the increase in crosslinking degree, the thermal shrinkage rate of the material at high temperatures significantly decreases. On one hand, compared to the non-crosslinked system, the foam material thickness in the crosslinked PP/POE blend system has increased, reaching a maximum value of 11.1 mm at a crosslinking degree of 12% and indicating a higher foaming ratio at this point. On the other hand, the thermal shrinkage rate of the crosslinked system has notably decreased, especially at a crosslinking degree of 55% where the shrinkage rate is only 13.4%, and the thickness after shrinkage remains at 8.4 mm, indicating good thermal stability. Thus, it is observed that an increase in the crosslinking degree significantly enhances the thermal stability of the material in the crosslinked system. Since foam materials often undergo thermal processing during fabrication, the thermal stability of the material is crucial. In the crosslinked system, this requirement is essentially met, and a higher crosslinking degree evidently contributes to improved thermal stability without compromising foaming efficiency.

##### Mechanical Properties

**Figure 7:**
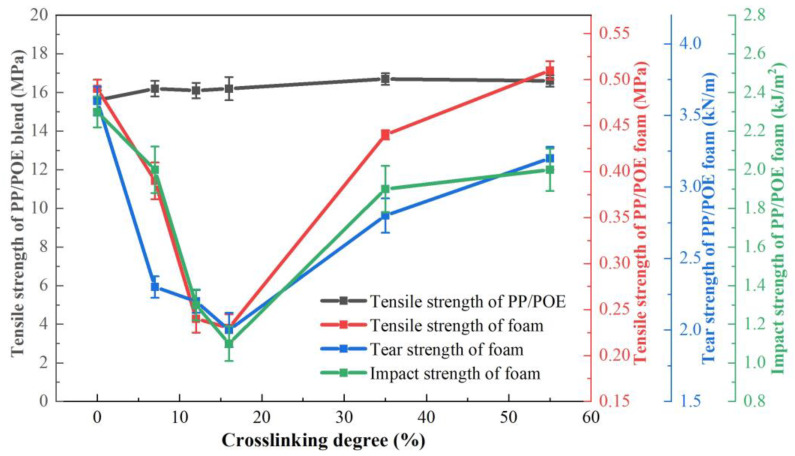
The tensile strength, tear strength, and impact strength of the PP/POE blend matrix and foam as a function of crosslinking degree.

The influence of the crosslinking degree on the tensile strength, tear strength, and impact toughness of the PP/POE blend system is shown in Figure 7. From Figure 7, which represents the change in tensile performance of the unfoamed PP/POE blend system, it is observed that the tensile strength of the crosslinked system is not significantly improved when compared to the non-crosslinked system. However, for the foamed system, an increase in crosslinking degree leads to a decrease followed by an increase in the tensile strength of foamed materials. At a crosslinking degree of 12%, the system exhibits the lowest tensile strength of 0.23 MPa, representing a 53% reduction compared to the non-crosslinked system. When the crosslinking degree reaches 55%, the tensile strength returns to 0.51 MPa. Thus, it can be inferred that the crosslinking degree itself has minimal impact on the tensile strength of unfoamed sheets. Instead, the tensile strength of foamed material is primarily determined by the foaming effect, i.e., foaming ratio and pore structure. Considering the SEM morphology in Figure 4, at a slight crosslinking degree of 12%, the pore size increases, foaming ratio increases, and pore density decreases, resulting in a loss of mechanical strength in the final foamed material. However, when the crosslinking degree is 55%, the foaming ratio decreases and the pore size is minimized, leading to an improvement in the tensile strength of the material. This phenomenon is also observed in tear strength and impact strength, exhibiting similar curves to the tensile strength variation, i.e., a decrease followed by an increase in strength. At a crosslinking degree of 12%, the tear strength and impact toughness are at their lowest, measuring 2.0 kN/m and 1.1 kJ/m^2^, respectively. As the crosslinking degree increases to 55%, tear strength and impact toughness rebound to 3.2 kN/m and 1.9 kJ/m^2^. This phenomenon is similar to the variation in tensile strength, influenced by the pore structure and pore size of the foamed material.

### 3.2. Fiber-Reinforced Crosslinked PP/POE Composite Foam

From the above results of irradiation crosslinking in the PP/POE blend matrix, it can be observed that mild crosslinking effectively enhances the melt strength of PP, significantly increases the foaming expansion ratio, reduces foam density, and improves high temperature thermal stability. However, it comes with a significant decrease in mechanical properties. Therefore, we further carried out fiber reinforcement modification on the aforementioned 12% crosslinked PP/POE blend system, corresponding to a 4 kGy irradiation dose in the experiment, to compensate for the adverse effects on mechanical properties caused by the high foaming expansion ratio.

#### 3.2.1. Impact of Fibers on Foaming Behavior

**Figure 8:**
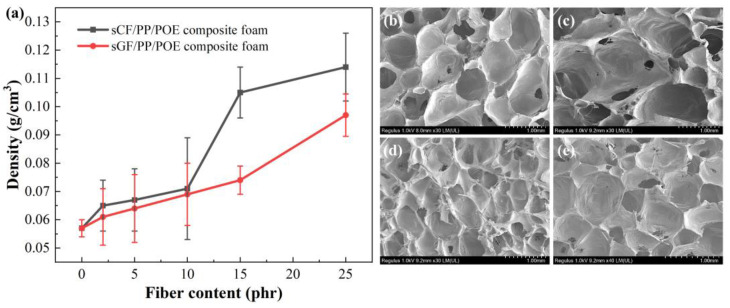
Effects of fiber content on (**a**) the PP/POE composite foam density, and pore cell morphology of (**b**,**c**) 10 phr- and 25 phr-reinforced sCF/PP/POE foam, and (**d**,**e**) 10 phr- and 25 phr-reinforced sGF/ PP/POE foam.

The density and cell morphology of fiber-reinforced irradiation-crosslinked PP/POE composite foams are shown in Figure 8. When the fiber content is less than 10 phr, there is no significant change in the foam density, only an increase of 10%. As the fiber content continues to increase beyond 15 phr, the density of fiber-reinforced crosslinked PP/POE composite foams sharply increases, and the foaming expansion ratio significantly decreases. Specifically, when the addition is 25 phr, the densities of sCF/PP/POE and sGF/PP/POE composite foams are 0.114 g/cm^3^ and 0.097 g/cm^3^, respectively, which have increased by 100% and 70%, respectively, compared to the PP/POE polymer foam without fiber addition at 0.057 g/cm^3^. This indicates that the foaming behavior of the crosslinked PP/POE blend system is significantly adversely affected at a high fiber content.

By examining the SEM cross-sectional morphology in Figure 8b–e of foams from 10 phr and 25 phr sCF/PP/POE and sGF/PP/POE composite materials, it can be observed that with 10 phr fiber addition, the cell morphology of both materials is relatively clear, and the cell structure is relatively uniform. However, compared to the system without fiber in Figure 4, the foaming structure of fiber-reinforced PP/POE composite materials is still somewhat damaged, showing some irregularly shaped cells and increased cell openings. With the addition of 25 phr fibers, the cell morphology of fiber-reinforced PP/POE composite materials is significantly affected, especially in the case of sCF/PP/POE composite foam, where the foam structure is severely disrupted with irregular cell walls and collapsing and merging phenomena occurring between cells.

#### 3.2.2. Impact of Fibers on Thermal and Mechanical Properties of Foam

##### Thermal Shrinkage Performance

**Figure 9:**
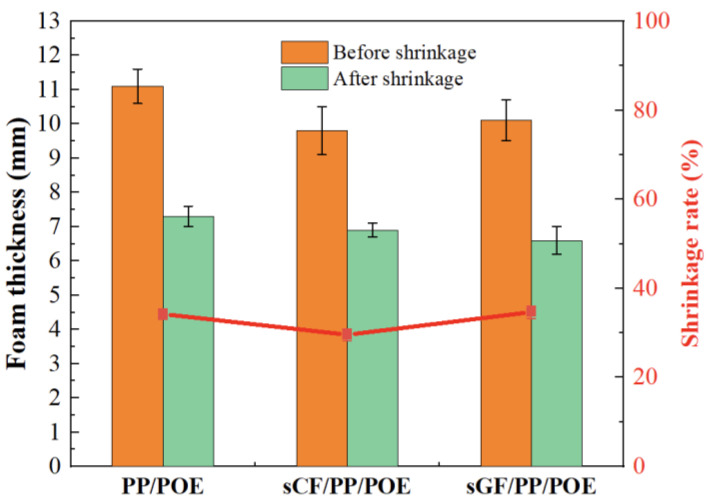
Impact of fibers on thermal shrinkage of PP/POE foam.

As shown in Figure 9, the addition of sCF is conducive to a slight reduction in the thermal shrinkage of PP/POE material, while sGF has almost no effect. This may be attributed to the negative thermal expansion coefficient of CF, which acts as a counterforce against certain degrees of thermal shrinkage. However, these changes are not significant. A possible reason is that although the addition of fibers is beneficial for increasing the thermal stability of thermoplastic polymers [51,52], in the case of foam structure, the shrinkage behavior is predominantly influenced by the continuous network structure dominated by polymers. Therefore, the contribution of fibers with a dispersed and low faction is limited. In addition, the introduction of fibers deteriorates the overall integrity of the foam and increases the pore size, leading to an increased probability of pore collapse after heating, offsetting the positive contribution of fibers. In the end, the thermal shrinkage stability is not significantly enhanced. However, despite the improvement in material viscosity and thermal shrinkage to some extent with the addition of carbon fibers in sCF/PP/POE, a further comparison with Figure 6 reveals that its contribution is far less significant than the enhanced thermal shrinkage stability brought about by irradiation crosslinking.

##### Mechanical Properties

**Figure 10:**
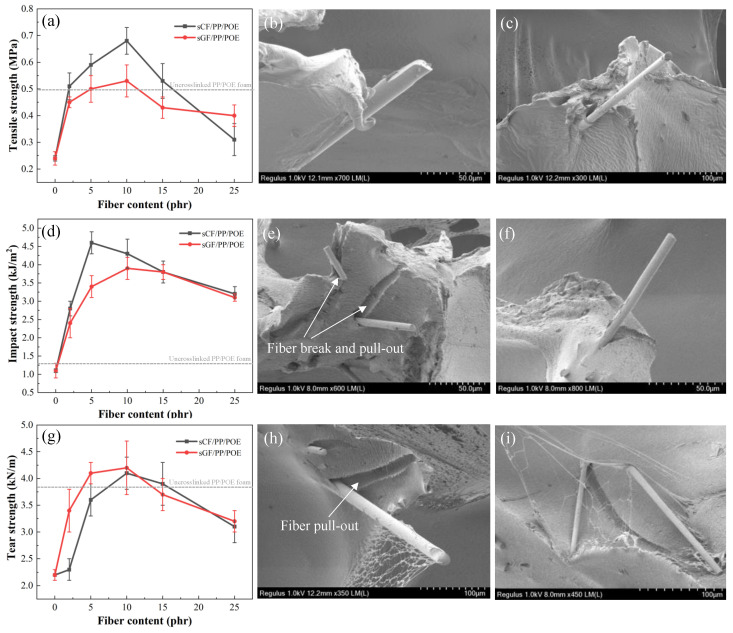
Effect of fiber content on the tensile behaviors of (**a**) tensile strength, SEM failure cross-section of crosslinked, (**b**) sGF/PP/POE composite foam, and (**c**) sCF/PP/POE composite foam; on the impact behaviors of (**d**) impact strength, SEM failure cross-section of crosslinked, (**e**) sGF/PP/POE composite foam, and (**f**) sCF/PP/POE composite foam; and on the tear behaviors of (**g**) tear strength, SEM failure cross-section of crosslinked, (**h**) sGF/PP/POE composite foam, and (**i**) sCF/PP/POE composite foam.

Figure 10a illustrates the relationship between tensile strength and fiber content for sCF/PP/POE and sGF/PP/POE composite foamed materials. It can be observed that the addition of sCF and sGF significantly enhances the tensile strength of PP/POE composite foamed materials, with both reaching their maximum values at 10 phr. However, the reinforcing effect of sCF is more pronounced than that of sGF. Specifically, at a sCF content of 10 phr, the tensile strength of the sCF/PP/POE composite foamed material increases to 0.69 MPa. This represents a 200% increase compared to the 0.23 MPa of the 12% crosslinked PP/POE system and a 41% increase compared to the 0.49 MPa of the non-crosslinked PP/POE system. This improvement is attributed to the uniform distribution of fibers within the pore walls in the crosslinked sCF/PP/POE composite foam system, which significantly enhances the polymer matrix. When the foamed material is loaded, fiber fracture and pull-out from the matrix can effectively absorb energy, increase the load required for material failure, and thereby enhance the mechanical properties of the foamed system (Figure 10b,c). For sCF, its individual fiber strength is higher than that of sGF, so the increase in pore wall strength is more pronounced after its addition. However, with a further increase in sCF content to 15 phr, a sharp decrease in tensile strength is observed for the sCF/PP/POE composite foamed material. At an sCF content of 25 phr, the tensile strength is only 0.29 MPa, approaching the mechanical strength of the system without added fibers. As shown in the SEM cross-sectional pore morphology in Figure 8, the addition of sCF at high content severely disrupts the internal pore structure, leading to significant loss of mechanical strength in the pores and ultimately resulting in a decrease in the overall mechanical strength of the material. This is because high fiber content often leads to increased matrix viscosity [46], hindering foaming, and it also introduces a large number of stress concentration points, making the cell walls prone to rupture and unable to form perfectly connected cell walls for load bearing. According to the classical theory *F* = *σA*, a large number of defects in the cell walls mean that the effective load-bearing area *A* decreases. Under the constant intrinsic strength *σ* of the material, the load-bearing capacity *F* decreases.

Similarly, with the increase in fiber content, both sCF/PP/POE and sGF/PP/POE composite foams exhibit a similar pattern of initially increasing and then decreasing impact strength (Figure 10d). However, there is a significant overall improvement in performance across the entire addition range. For sCF, the impact strength reaches the maximum value of 4.5 kJ/m^2^ at a content of 5 phr. This represents a 246% increase compared to the 1.3 kJ/m^2^ of the 12% crosslinked PP/POE system and a 96% increase compared to the 2.3 kJ/m^2^ of the non-crosslinked PP/POE system. As for sGF, its impact strength reaches the maximum value of 3.6 kJ/m^2^ at a content of 10 phr. Similarly to the tensile process, the impact fracture failure process is accompanied by fiber fracture and pull-out, so the reinforcing effect of sCF is better than that of sGF.

Figure 10g shows the variation curve of the tear strength of sCF/PP/POE and sGF/PP/POE composite foamed materials with fiber content. It can be observed that the tear strength of PP/POE composite foamed materials is significantly improved with the addition of sCF and sGF, and both start to decrease after the addition exceeds 10 phr. When comparing the two fibers, the enhancement of tear strength by sGF is more pronounced, reaching a maximum tear strength of 4.2 kN/m. There is no obvious fiber fracture from the fractured surface morphology after tearing, and the main failure mode is the debonding at the fiber/matrix interface and fiber pull-out. Due to the better interface compatibility of sGF with the matrix, compared to the unmatched epoxy sizing of sCF, sGF shows a more pronounced enhancement in tear strength.

In summary, simultaneously combining the results from this section with those of Section 3.1, crosslinking modification has a significant enhancing effect on the melt strength and high temperature thermal stability of PP/POE foam, with little impact on mechanical properties. Conversely, fibers have a significant impact on improving mechanical properties, with little effect on melt strength and high temperature thermal stability. In the fiber-reinforced crosslinked PP/POE blend foam system, the two mechanisms achieve a good complementary effect.

## 4. Conclusions

This study addresses the challenge of achieving high foaming ratios and high performance of polypropylene (PP) foam, caused by its low melt strength. A systematic exploration of material composition, irradiation crosslinking, and fiber reinforcement was conducted. Ultimately, a fiber-reinforced PP/POE composite foam with both low-density and high-performance characteristics was obtained. The main conclusions are as follows:

(1) The crosslinking degree of the PP/POE blend can be effectively controlled within the range of 0–60% by adjusting the irradiation dose and crosslinking agent content. The crosslinking degree is positively correlated with the irradiation dose and crosslinking agent content, while it exhibits an increase and then decrease trend with the foaming ratio. At a crosslinking degree of 12%, the foaming ratio of the crosslinked PP/POE blend can reach 16, representing a 36% reduction in density and a 60% increase in foaming ratio compared to the non-crosslinked PP/POE blend.

(2) Mild crosslinking at 12% effectively enhances the melt strength of PP, significantly improves the foaming ratio, reduces foam density, and enhances resistance to high temperature thermal shrinkage. However, it leads to a 53% decrease in mechanical properties. Through further fiber reinforcement, the mechanical strength of the crosslinked PP/POE composite material is significantly improved. Specifically, the foam density of 10 phr carbon-fiber-reinforced crosslinked sCF/PP/POE composite material is comparable to that of the 12% crosslinked PP/POE system, but the tensile strength reaches 0.69 MPa, increasing by 200% compared to the 12% crosslinked PP/POE system and by 41% compared to the non-crosslinked PP/POE system. It also exhibits a 96% increase in impact strength and an 11% increase in tear resistance when compared to the non-crosslinked PP/POE system.

(3) In summary, irradiation crosslinking is beneficial for enhancing the melt strength and resistance to high temperature thermal shrinkage of PP/POE foam, while fiber reinforcement contributes significantly to improving mechanical properties. These achieve a good complementary effect in low-density and high-performance PP foam modification.

## Figures and Tables

**Table 1:** The composition of PP/POE blend and fiber-reinforced PP/POE composite foams.

Material	PP	POE	Nucleating Agent	Lubricating Agent	Antioxygen Agent	Foaming Agent	Crosslinking Agent	Reinforced Fibers
Brand	S131	6202	TMA-3	Stearic acid	1076G	ADC	TMPTMA	sCF or sGF
Content (g)	60	40	0.4	1.0	0.5	15	0–16	0–20

## Data Availability

Data are contained within the article.
